# Subspecies Typing of *Streptococcus agalactiae* Based on Ribosomal Subunit Protein Mass Variation by MALDI-TOF MS

**DOI:** 10.3389/fmicb.2019.00471

**Published:** 2019-03-11

**Authors:** Julian Rothen, Joël F. Pothier, Frédéric Foucault, Jochen Blom, Dulmini Nanayakkara, Carmen Li, Margaret Ip, Marcel Tanner, Guido Vogel, Valentin Pflüger, Claudia A. Daubenberger

**Affiliations:** ^1^Department of Medical Parasitology and Infection Biology, Swiss Tropical and Public Health Institute (Swiss TPH) Basel, Basel, Switzerland; ^2^University of Basel, Basel, Switzerland; ^3^Research Group for Environmental Genomics and Systems Biology, Institute of Natural Resource Sciences, Zurich University of Applied Sciences (ZHAW), Wädenswil, Switzerland; ^4^Mabritec AG, Riehen, Switzerland; ^5^Bioinformatics and Systems Biology, Justus-Liebig-Universität Gießen, Giessen, Germany; ^6^Department of Microbiology, The Chinese University of Hong Kong, Shatin, Hong Kong

**Keywords:** group B *Streptococcus*, MALDI-TOF, mass spectrometry, ribosomal subunit protein, molecular epidemiology, bacterial typing

## Abstract

**Background:** A ribosomal subunit protein (rsp)-based matrix-assisted laser desorption/ionization time-of-flight mass spectrometry (MALDI-TOF MS) method was developed for fast subspecies-level typing of *Streptococcus agalactiae* (Group B *Streptococcus*, GBS), a major cause of neonatal sepsis and meningitis.

**Methods:** A total of 796 GBS whole genome sequences, covering the genetic diversity of the global GBS population, were used to *in silico* predict molecular mass variability of 28 rsp and to identify unique rsp mass combinations, termed “rsp-profiles”. The *in silico* established GBS typing scheme was validated by MALDI-TOF MS analysis of GBS isolates at two independent research sites in Europe and South East Asia.

**Results:** We identified *in silico* 62 rsp-profiles, with the majority (>80%) of the 796 GBS isolates displaying one of the six rsp-profiles 1–6. These dominant rsp-profiles classify GBS strains in high concordance with the core-genome based phylogenetic clustering. Validation of our approach by in-house MALDI-TOF MS analysis of 248 GBS isolates and external analysis of 8 GBS isolates showed that across different laboratories and MALDI-TOF MS platforms, the 28 rsp were detected reliably in the mass spectra, allowing assignment of clinical isolates to rsp-profiles at high sensitivity (99%) and specificity (97%). Our approach distinguishes the major phylogenetic GBS genotypes, identifies hyper-virulent strains, predicts the probable capsular serotype and surface protein variants and distinguishes between GBS genotypes of human and animal origin.

**Conclusion:** We combine the information depth of whole genome sequences with the highly cost efficient, rapid and robust MALDI-TOF MS approach facilitating high-throughput, inter-laboratory, large-scale GBS epidemiological and clinical studies based on pre-defined rsp-profiles.

## Introduction

*Streptococcus agalactiae* (Group B *Streptococcus*, GBS), a gram-positive bacterium colonizing the gastrointestinal and urogenital tract of around 18% of pregnant women worldwide (Russell et al., 2017), is a leading cause of neonatal and early infant sepsis and meningitis. It has been estimated that in 2015, GBS caused around 90,000 deaths in infants under the age of 3 months and 57,000 cases of fetal infections/stillbirths (Seale et al., 2017). The reason for the emergence of GBS as an important human pathogen has been attributed to the spread of pathogenic GBS clones (Sørensen et al., 2010) mainly driven by the widely use of tetracycline, resulting in globally established, genetically homogeneous GBS lineages in humans (Da Cunha et al., 2014). In contrast, obligate animal GBS strains, which are often species-specific, represent an under-researched reservoir of genetically highly diverse genotypes with zoonotic potential (Fischer et al., 2013; Godoy et al., 2013).

GBS carry polysaccharide capsules which are main virulence factors interfering with phagocytic clearance of the bacteria (Chen et al., 2013). Ten GBS capsular serotypes (Ia, Ib, II, III, IV, V, VI, VII, VIII, IX) have been described and are used to classify GBS and to monitor population dynamics (Slotved et al., 2007). Distinct serotypes are associated with higher virulence, in particular serotype III, but also serotypes Ia, Ib and V, which together account for the majority of invasive disease cases in infants (Seale et al., 2017). Multi-locus sequence typing (MLST) has also been developed and applied for GBS discrimination. According to MLST, global GBS populations are dominated by five major clonal complexes (CC), namely CC1, CC10, CC17, CC19, and CC23 with CC17, consisting mainly of serotype III strains, being most pathogenic (Jones et al., 2003).

Vaccination of pregnant women during the second and third trimester has been proposed as an approach to prevent GBS disease in both mothers and infants (Baker et al., 2003). A trivalent glycoconjugate vaccine, covering serotypes Ia, Ib, and III has completed phase I and II clinical trials (Heyderman et al., 2016; Leroux-Roels et al., 2016) and a pentavalent vaccine including serotypes Ia, Ib, II, III, and V is under development (Kobayashi et al., 2016). Protein based vaccines that target surface antigens (pili and alpha-like proteins) are also developed but they will require to overcome amino acid sequence variation to induce GBS strain cross-protection (MinervaX, 2017; Nuccitelli et al., 2011). Consequences of vaccine deployment on GBS population dynamics needs to be monitored closely, following possible capsular switches, strain replacements or emergence of novel GBS strains (Bellais et al., 2012; Kobayashi et al., 2016).

Matrix-assisted laser desorption/ionization time-of-flight mass spectrometry (MALDI-TOF MS) has become the gold standard for high-throughput microbial species identification in clinical settings (Seng et al., 2009; Singhal et al., 2015). Commercially available and validated MALDI-TOF MS systems rely on detection of generic peptide patterns, limiting the discriminatory power for closely related species and separation of subspecies (van Belkum et al., 2017; Body et al., 2018). The conserved ribosomal subunit proteins (rsp) are cytosolic proteins of high abundance and in the molecular weight range detectable by commercial MALDI-TOF MS equipment. Whole genome sequence (WGS) data can be used to *in silico* predict molecular masses of distinct rsp. These rsp masses can then be measured by MALDI-TOF MS, thereby providing a targeted, biomarker-based approach of classifying mass spectra, superior to the conventional “pattern-recognition” approach (Suarez et al., 2013; Ziegler et al., 2015). Here, a highly cost efficient, rapid and robust rsp-based MALDI-TOF MS GBS typing approach has been developed that is transferable between laboratories and enables large-scale GBS epidemiological and clinical studies.

## Results

### Capsular Serotype and Sequence Type Distribution in Global GBS Collection

A total of 796 GBS WGS was collated from public databases and in-house sequenced isolates from human, camel, bovine and other animal origin. A listing of the 796 GBS isolates and their corresponding metadata is provided in Supplementary Table S1. *In silico* MLST revealed the presence of 108 sequence types (ST) and except for ST327, all ST that are among the 28 most abundant in the global population were present (Supplementary Figure S1). In accordance with the PubMLST *S. agalactiae* isolate database (Jolley and Maiden, 2010), ST17, ST1, ST23 and ST19 were the most frequent ST, accounting for 36% isolates in our collection (50% in PubMLST). All capsular serotypes, except serotype VII, were represented with Ia (*n* = 126), Ib (*n* = 52), II (*n* = 211), III (*n* = 183), IV (*n* = 80), V (*n* = 111), VI (*n* = 9), VIII (*n* = 1), IX (*n* = 2) and non-type-able (*n* = 21). In summary, these 796 WGS constitute a representation of the known global GBS population.

### Genome-Wide Phylogenetic Analysis of GBS Collection

**FIGURE 1:**
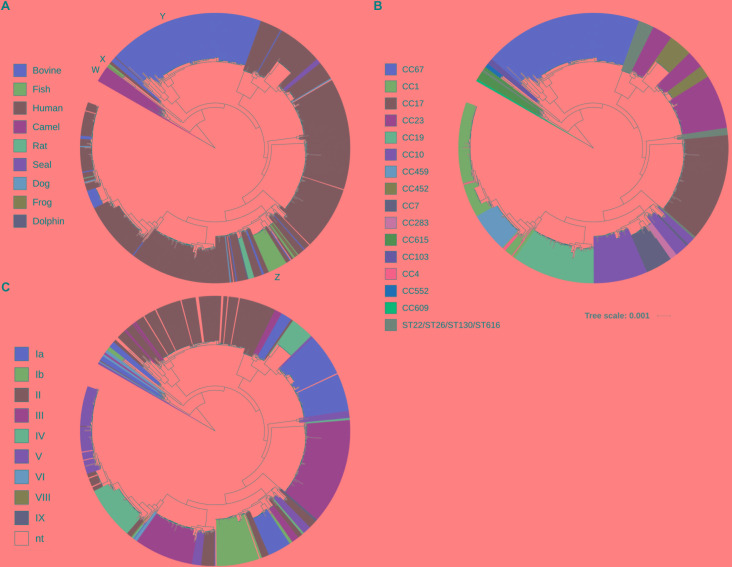
Fast Tree phylogenetic tree based on core-genome analysis of 796 Group B *Streptococcus* whole genome sequences. Individual strains are annotated with **(A)** host origin, **(B)** *in silico* predicted multi-locus sequence typing clonal complex (CC) or sequence type (ST) and **(C)** *in silico* predicted capsular serotype. (Scale bar: nucleotide substitutions per site). The capital letters in **(A)** mark phylogenetically distinct clusters of obligate camel (W), fish/frog (X) or cattle (Y) origin and one heterogeneous cluster (Z) consisting of GBS strains from various hosts.

The evolutionary relationship of these 796 GBS strains was then assessed by core-genome based phylogenetic analysis. Sixteen GBS strains originating from *Camelus dromedarius* were found distant from all other GBS strains (Figure 1A, marked as W). Other isolates from fish and frog (Figure 1A, marked as X) and cattle (Figure 1A, marked as Y) formed distinct, host origin specific phylogenetic clusters. The other phylogenetic clusters consisted predominantly of GBS of human origin, with sporadic presence of animal associated strains. An exception to this was one distinct phylogenetic cluster containing strains of bovine, dog, fish, human and rat origin (Figure 1A, marked as Z).

Core-genome based phylogenetic clustering was also compared with *in silico* classification based on MLST. A total of 108 sequence types (ST) were grouped into 15 clonal complexes (CC) of closely related isolates (CC67, CC1, CC17, CC23, CC19, CC10, CC459, CC452, CC7, CC283, CC615, CC609, CC103, CC4, and CC552). A high agreement between MLST classification of GBS isolates and the core-genome phylogenetic clustering was obvious (Figure 1B). As expected, core-genome based classification provides better resolution, thereby further sub-grouping genotypes that appear identical by MLST. In some cases, the genetic variation of such sub-groups puts them into overall closer phylogenetic relationship with genotypes of other CC like CC23 and CC452, CC10, CC7 and CC283 or CC1, CC4 and CC459 (Figure 1B). Comparison of core-genome phylogenetic clustering with *in silico* assigned capsular serotypes confirmed that genotypes clustering together are likely to share identical capsular serotypes (Figure 1C). Expression of variants of five GBS surface proteins including alpha-like protein (Alp) gene family, pilus islands, surface immunogenic protein (Sip), laminin-binding protein (Lmb) and Group B *Streptococcus* immunogenic bacterial adhesin (BibA) followed largely core-genome based phylogeny (Supplementary Figure S2).

### Assessment of Measurability and Variability of *in silico* Predicted Ribosomal Subunit Protein Molecular Masses

**FIGURE 2:**
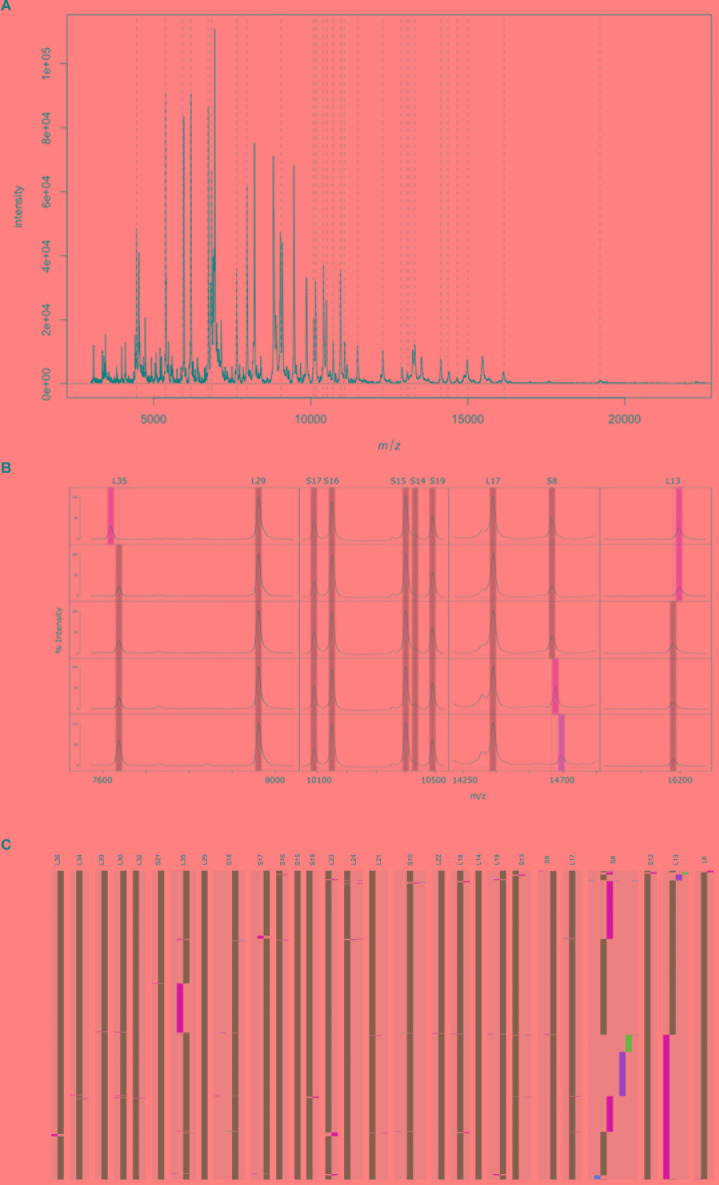
A representative MALDI-TOF mass spectrum of a *Streptococcus agalactiae* ST7 strain, covering the mass range between 4,000 Da and 22,000 Da. The arbitrary intensity values of the mass peaks are given on the *y*-axis. Dashed lines indicate the position of the 28 ribosomal subunit proteins (rsp) targeted in our analyses. **(B)** Assessment of mass spectra belonging to five Group B *Streptococcus* (GBS) isolates confirm *in silico* predicted mass shifts in three rsp (L35, L13, and S8). Green: Major rsp mass, yellow and orange: rsp mass variants. **(C)** *In silico* predicted molecular mass variation of 28 rsp across 796 GBS whole genome sequences. Ribosomal subunits proteins are ordered from left to right by increasing molecular weight. Green: Most abundant rsp mass allele; yellow, orange and blue: 2nd, 3^rd^, and 4th most abundant rsp mass allele; Red: remaining rsp mass alleles.

Whole genome sequence data of 29 GBS isolates available and cultured under in-house conditions were used to predict *in silico* molecular masses of all known 59 rsp. MALDI-TOF MS analysis conducted with these 29 GBS isolates revealed that 28 rsp were reproducibly measured in a molecular weight range between 4,425 Da and 19,293 Da (Figure 2A). These experiments confirmed that our novel sample preparation protocol enabled us to identify mass variation among 28 distinct rsp (Figure 2B). Next, the remaining 767 WGS were used to predict *in silico* molecular masses of these 28 rsp, including S8–S10, S12, S13, S15–S19 and S21 of the small ribosomal subunit and L6, L13, L14, L17-L19, L21-L24, L29, L30, and L32-L36 of the large ribosomal subunit. Three rsp (L14, L29, and S15) did not show allelic mass variation across all 796 isolates. Four rsp (L22, L32, L33 and S21) showed a variant mass in 1 out of 796 isolates. Eighteen rsp (L6, L17-L19, L21, L23, L24, L30, L34, L36, S9, S10, S12, S13, and S16-S19) showed mass variants in fewer than 8 out of 796 isolates. The most variable rsp were L13, L35, and S8 displaying mass variation in > 100 out of 796 isolates (Figure 2C).

### Definition of rsp-Profiles in GBS Collection

**FIGURE 3:**
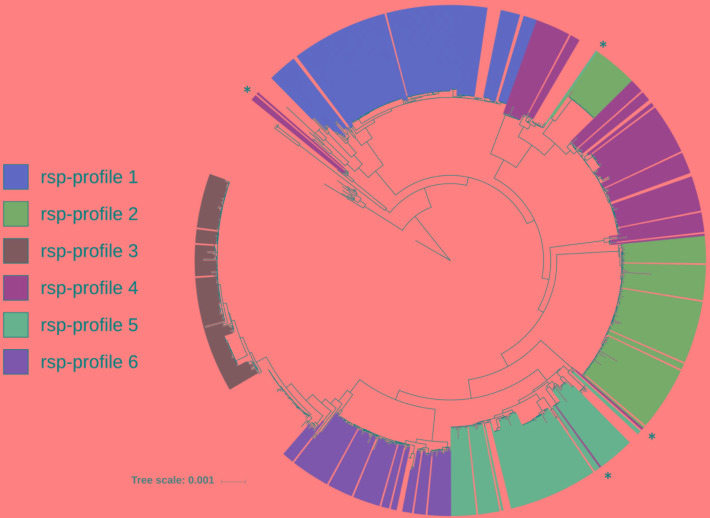
FastTree phylogenetic tree based on core-genome analysis of 796 Group B *Streptococcus* whole genome sequences (WGS). Individual strains are annotated with their *in silico* determined ribosomal subunit proteins (rsp)-profile. For simplicity, only the six globally dominant rsp-profiles are shown (covering 83% of isolates in our WGS collection). Marked with asterisks are eight strains whose rsp-profile was miss-assigned due to limitation of MALDI-TOF MS resolution (i.e., 400 ppm). (Scale bar: nucleotide substitutions per site).

**FIGURE 4:**
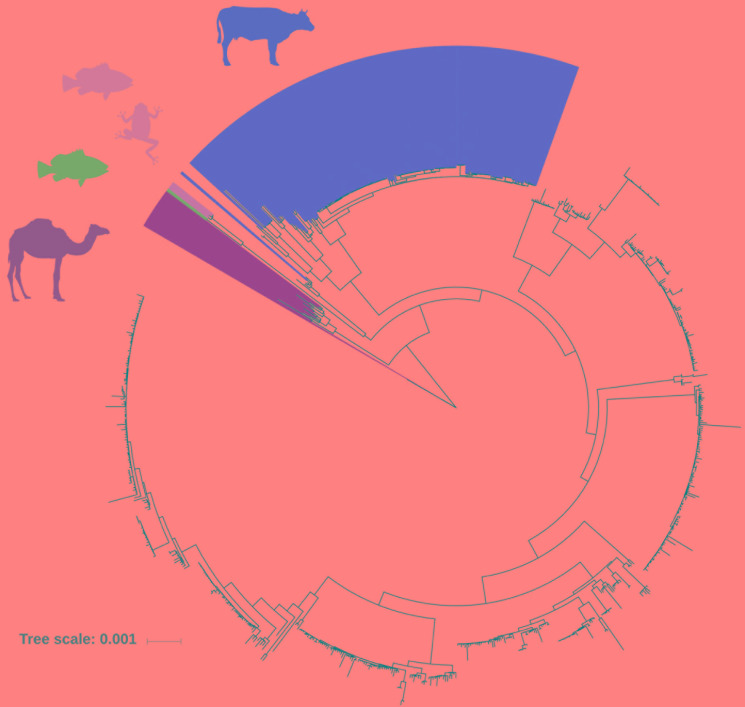
FastTree phylogenetic tree based on core-genome analysis of 796 Group B *Streptococcus* (GBS) whole genome sequences. Individual GBS strains are annotated with ribosomal subunit proteins (rsp)-profiles, which are distinct for GBS genotypes of obligate animal origin. Red: rsp-profiles 1, 10, 12, 17, 19, 38, 39, 59, 60, and 61 are exclusively found in bovine isolates. Blue: rsp-profile 49 is unique for fish origin. Rose: rsp-profile 13 is indicative of either fish or frog origin. Khaki: rsp-profiles 11, 15, 18, 20, 21, and 37 are exclusively found in camel isolates. (Scale bar: nucleotide substitutions per site).

Next, we predicted all possible combinations of these 28 rsp based on the 796 GBS WGS collection. Limited by the MALDI-TOF MS detection accuracy (400 ppm threshold), 62 unique combinations of the 28 rsp were identified, which are referred to as rsp-profiles. Six dominant rsp-profiles (rsp-profiles 1–6), present in 83 to 134 GBS isolates covered 83% of the isolates (657/796). Five rsp-profiles (rsp-profiles 7–11) were present in 5 to 42 GBS representing 9% (72/796) of isolates. Rsp-profiles 12–22 existed in 2 to 4 isolates (27/796, 3%) and the remaining 40 rsp-profiles named 23–62 were singletons (40/796, 5%) (Supplementary Figure S3). Rsp-profiles classified GBS strains in high concordance with the core-genome based phylogenetic clustering (Figure 3). GBS strains sharing an identical rsp-profile were located either next to each other or in the same subordinate cluster in the core-genome based phylogenetic tree. Exceptions were one rsp-profile 5 and two rsp-profile 4 strains that were grouped to strains with different rsp-profiles as well as five ST103 strains with rsp-profile 4 that formed a separate group in the core-genome analysis (Figure 3). These strains displayed novel, *in silico* predicted, rsp-profiles but the molecular mass differences to rsp-profile 4 and rsp-profile 5, respectively, were undetectable by our MALDI-TOF MS, highlighting the technical limitations of the mass spectrometer. GBS strains isolated from camels displayed a large variety of camel-specific rsp-profiles. Unique, animal-specific rsp-profiles were also observed in ST260/ST552 strains originating from frogs (*n* = 1) and fish (*n* = 3) and in CC67, ST61, ST591 and ST622 (*n* = 150) bovine strains (Figure 4 and Supplementary Table S1).

### Co-occurrence of rsp-Profiles With MLST, Capsular Serotyping and Surface Protein Expression

**FIGURE 5:**
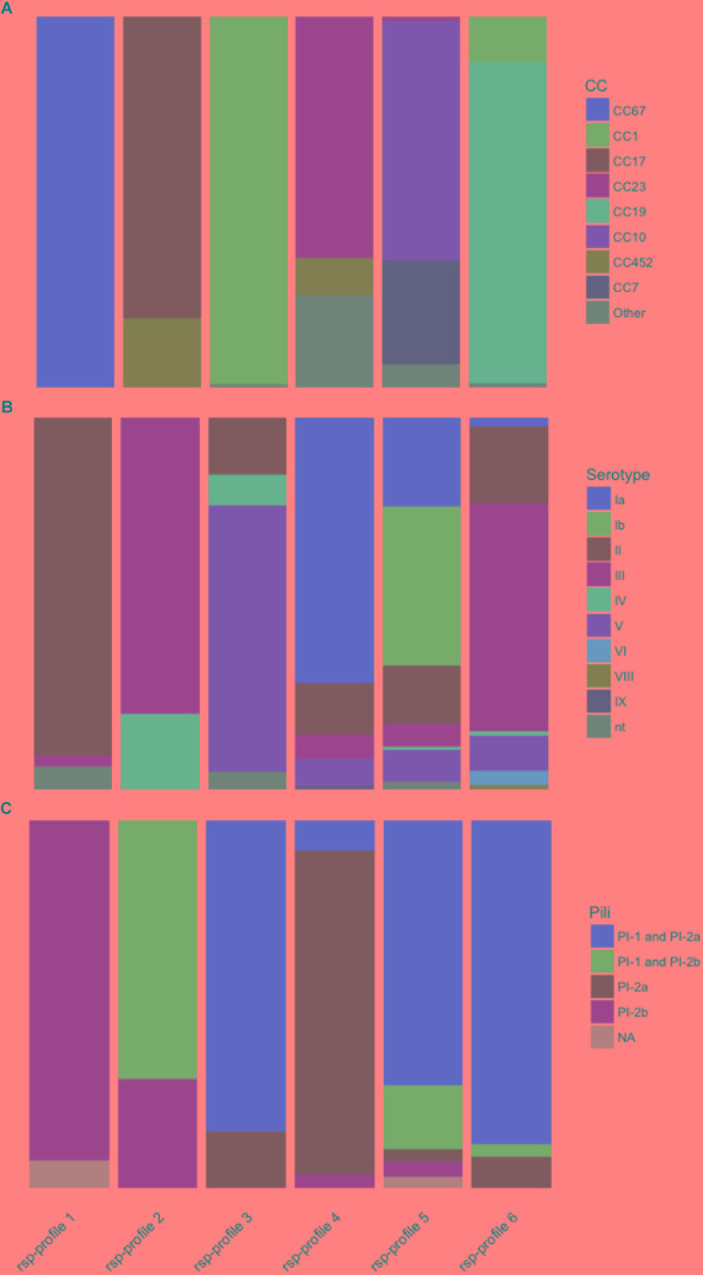
The global major ribosomal subunit proteins (rsp)-profiles 1–6 provide a probabilistic value regarding the Group B *Streptococcus* genotypes’ associated **(A)** multi locus sequence typing clonal complex (CC), **(B)** capsular serotype and **(C)** pilus variants. nt, non-typeable.

We investigated how GBS strains belonging to the six dominant rsp-profiles compared to *in silico* predicted MLST based CC (Figure 5A), capsular serotypes (Figure 5B) and pilus island variants (Figure 5C) in our GBS collection. We found that the rsp-profile of a given GBS strain provided a reliable predictive value regarding its membership to a distinct CC, expression of capsular serotype and pilus island variants (Supplementary Table S2).

### Whole-Cell Lysate MALDI-TOF MS Analysis of GBS

A total of 248 GBS isolates were grown and then analyzed in quadruplicates by MALDI-TOF MS resulting in 992 mass spectra. Twenty-five rsp were detected in > 98% of all spectra acquired. L34, L6, and L33 were found in 96, 91 and 80% of mass spectra acquired, respectively. For the 29 GBS isolates grown in-house and with WGS available, 14 distinct rsp-profiles, including rsp-profiles 2–6, 11, 15, 18–22, 37, and 55 were predicted *in silico*. Validation by MALDI-TOF MS demonstrated 100% sensitivity, with all 29 GBS being assigned an rsp-profile (Table 1A). All but one of the MALDI-TOF MS measured rsp-profiles corresponded to the *in silico* predicted rsp-profiles (specificity of 97%). One isolate, classified as rsp-profile 6, should display the unique rsp-profile 55 according to *in silico* prediction. Rsp-profile 55 differs by an unique mass allele of rsp L32 from rsp-profile 6 and we cannot exclude the possibility that lack of nucleotide sequence quality led to dubious *in silico* prediction of L32.

**Table 1:** Validation of the established ribosomal subunit protein (rsp) typing scheme with MALDI-TOF MS measurements of in-house group B *Streptococcus* (GBS) isolates.

(A) MALDI-TOF MS measurements of 37 isolates with available whole genome sequence (WGS) data.				

***n* isolates**	**source**	**MALDI assigned rsp-profile**	***in silico* assigned rsp-profile**	***n* correctly assigned**
3	Human	rsp-profile 2	rsp-profile 2	3
1	Human	rsp-profile 3	rsp-profile 3	1
1	Human	rsp-profile 4	rsp-profile 4	1
3	Human	rsp-profile 5	rsp-profile 5	3
1	Human	rsp-profile 6	rsp-profile 6	1
5	Camel	rsp-profile 11	rsp-profile 11	5
3	Camel	rsp-profile 15	rsp-profile 15	3
2	Camel	rsp-profile 18	rsp-profile 18	2
2	Bovine	rsp-profile 19	rsp-profile 19	2
2	Camel	rsp-profile 20	rsp-profile 20	2
2	Camel	rsp-profile 21	rsp-profile 21	2
1	Camel	rsp-profile 22	rsp-profile 22	1
1	Human	rsp-profile 22	rsp-profile 22	1
1	Camel	rsp-profile 37	rsp-profile 37	1
1	Human	rsp-profile 6	rsp-profile 55	0
8 ^∗^	Fish	rsp-profile 5	rsp-profile 5	8

**37/37 assigned (100% sensitivity)**				**specificity: 97% (36/37)**

**(B)** MALDI-TOF MS measurements of 219 isolates without WGS available.				

***n* assigned**	**source**	**MALDI assigned rsp-profile**		
33	Human	rsp-profile 2		
16	Human	rsp-profile 3		
49	Human	rsp-profile 4		
22	Human	rsp-profile 5		
30	Human	rsp-profile 6		
1	Human	rsp-profile 7		
22	Camel	rsp-profile 11		
20	Camel	rsp-profile 15		
17	Camel	rsp-profile 18		
2	Bovine	rsp-profile 19		
2	Camel	rsp-profile 20		
1	Camel	rsp-profile 21		
1	Camel	rsp-profile 37		
0 (3)^∗∗^	Human	new profile		

**216/219 assigned (99% sensitivity)**		

^*^Isolates typed in an external laboratory on a different MALDI-TOF MS system. ^∗∗^Three isolates displayed novel rsp-profiles not yet contained in our reference database.

For 219 GBS isolates without WGS available, rsp-profiles were assigned to 210/219 isolates (Table 1B). Manual inspection of the spectra of six unassigned isolates revealed that some rsp were missed during automated assignment due to poor resolution of their mass peaks. These rsp were updated accordingly and the six isolates could subsequently be assigned to an rsp-profile, producing an overall sensitivity of 99% (216/219 isolates assigned a rsp-profile). A total of 150 GBS isolates were assigned to the dominating rsp-profiles 2–6. Of the GBS isolates originating from cows, five ST591 strains were assigned to rsp-profile 19 and one strain (SLV ST19) was assigned to rsp-profile 6. Sixty-five isolates of camelid origin were assigned to seven different rsp-profiles that are, with exception of rsp-profile 22, specific for camel genotypes. Three isolates could not be classified because they displayed an rsp-profile not yet contained in our reference database based on the 796 WGS collection.

### Confirmation of Inter-Laboratory Reproducibility of Our Method

Matrix-assisted laser desorption/ionization time-of-flight mass spectrometry typing of eight GBS isolates of fish origin confirmed that our rsp-based typing method is transferable to other research sites and MALDI TOF MS equipment. Sample processing following our protocol and measurement on a Microflex MALDI-TOF MS produced high quality mass spectra, with all 28 rsp detected. All isolates were assigned to rsp-profile 5, one of the global dominant lineages. *In silico* prediction of the 28 rsp molecular masses based on the available WGS confirmed the correct identification for all eight isolates (Table 1A).

## Discussion

We have harnessed the advantages of information depth generated by WGS with the highly cost efficient, rapid and robust MALDI-TOF MS approach to develop a high-throughput, biomarker-based typing method for GBS clinical and epidemiological research. GBS typing methods based on MLST, pulsed-field gel electrophoresis (PFGE) or capsular serotyping can provide insight into GBS epidemiology. However, a significant proportion of non-typeable strains cannot be identified by capsular serotyping and PFGE results are difficult to compare across laboratories. These methods are also limited due to their time-consuming nature, considerable per sample processing costs and in inferring evolutionary relationships between strains (Furfaro et al., 2018). MALDI-TOF MS has become a recent standard for clinical microbiological diagnostics (Seng et al., 2009; Singhal et al., 2015) and it has been applied to identify GBS hyper-virulent ST17 and ST1 strains based on single biomarker masses of either unknown (Lartigue et al., 2011) or non-rsp identity (Lin et al., 2017).

Rapidly increasing availability of WGS data with development of computational analysis tools allows now in-depth comparison of whole bacterial genomes (Tettelin et al., 2005). We used 796 GBS WGS that were representative of the global GBS population and developed a MALDI-TOF MS based GBS typing method resting on detection of allelic mass differences of pre-defined markers. Our approach targets 28 distinct rsp with a molecular mass between 4,425 Da and 19,293 Da, allowing us to simultaneously detect molecular mass variation across a concatenated amino acid sequence of ∼2,700 amino acids. A refined sample preprocessing protocol was developed enabling high-throughput analyses of GBS samples yielding high-level spectra quality. Analysis of one isolate takes less than an hour and can easily be up-scaled, permitting typing of 40–60 isolates daily. Our newly developed bioinformatics analysis pipeline requires minimal bioinformatics knowledge and hands on time by routine users. Per sample analysis costs < 6 USD renders our approach highly competitive to currently employed GBS identification methods.

We identified six dominant rsp-profiles that classify GBS genotypes in high concordance to their corresponding core-genome phylogeny (Figure 3). Comparing these six rsp-profiles against 115,768 MALDI-TOF MS spectra obtained from routine diagnostic analysis and covering 3,013 bacterial species revealed that the best matching species following GBS was *Streptococcus pyogenes* with only 15/28 rsp detected. This demonstrates the high specificity and discriminatory power of our rsp-profile based typing.

One limitation of our method is that only strains with rsp-profiles deposited in the reference database can be classified unequivocally. In case of GBS strains with an unknown rsp-profile, our method will flag these strains for later conduct of WGS. The *in silico* extracted novel rsp-profiles can then be incorporated into an updated reference database for typing of this novel strain. Another limitation rests with the inherent technical capacity of MALDI-TOF MS to discriminate rsp masses. Minimal molecular weight differences of less than 400 ppm are below the detection threshold of a routine MALDI-TOF MS machine. This can lead to failure in distinguishing certain rsp-profiles with false assignment of rsp-profiles. Future advances in MALDI-TOF MS technology, improving both accuracy and overall mass range coverage will help to overcome this.

We propose several possible applications for the rsp-based MALDI-TOF MS typing method: (i) Identification of hyper virulent CC17 and CC23 strains and related ST452 (Campisi et al., 2016). (ii) Tracking of potential zoonotic GBS since most strains of animal origin display different rsp-profiles than human GBS strains (rsp-profiles 1, 10, 12, 17, 19, 38, 39, 59, 60, and 61 are confined to bovine origin, rsp-profile 49 is unique for fish origin, rsp-profile 13 is indicative of either fish or frog origin and rsp-profiles 11, 15, 18, 20, 21, and 37 are unique for cameloid origin (Figure 4). (iii) First-line screening for specific, disease-causing strains as for the GBS disease outbreak in Singapore affecting adult populations (Kalimuddin et al., 2017). (iv) Monitoring of post-vaccine introduction impact on GBS population structure. As learned with the 7-valent and 13-valent pneumococcal vaccines, the introduction of a glycoconjugate vaccine targeting only a fraction of circulating serotypes can lead to the emergence of non-vaccine-type strains associated with clinical disease (Miller et al., 2011; Weinberger et al., 2011; Devine et al., 2017). A similar scenario might occur after introduction of a trivalent GBS glycoconjugate vaccine, highlighting the importance of large-scale GBS population monitoring. Given that an assigned rsp-profile provides a robust predictive value regarding capsular serotype (Figure 5), our method could be used to monitor if rsp-profiles carrying the vaccine-type serotypes Ia, Ib, and III vanish over time while other rsp-profiles linked to non-vaccine-type serotypes increase.

In summary, our approach and database is flexible for inclusion of novel rsp-profiles, robust against inter-laboratory variation of mass spectra quality, streamlined for easy application by minimally trained users, suitable for high-throughput, large scale GBS epidemiological and clinical studies and highly cost efficient with per sample analysis costs of < 6 USD and results obtained within minutes.

## Materials and Methods

### GBS WGS Used for *in silico* Analyses

A total of 876 WGS were obtained on 24-07-2017 from the National Center for Biotechnology Information (NCBI) genome database. A subset of 98 WGS was removed from the dataset since sequence quality did not allow prediction of all 28 rsp molecular masses. Together with 18 in-house sequenced isolates, the final dataset consisted of 796 WGS. The dataset contained data from GBS genotypes isolated over a long time span, with collection time points dating from 1934 to 2016. The strains stem from various geographic regions in Africa, the Americas, Asia, Australia, and Europe. The majority of the strains were isolated from human (*n* = 543) or cattle (*n* = 187), with the remaining genotypes isolated from fish (*n* = 25), camel (*n* = 16), rat (*n* = 7), seal (*n* = 5), dog (*n* = 4), frog (*n* = 2), dolphin (*n* = 1), or unknown origin (*n* = 6). Of the strains for which information of the host health was available, 449 were reported to be associated with disease, while 277 occurred as non-disease-causing colonizer. Comprehensive metadata of all WGS is provided in Supplementary Table S1.

### GBS Whole-Genome Sequencing

Genomic DNA was extracted using the QIAamp DSP DNA minikit (Qiagen, Hilden, Germany). A first batch of three isolates was processed as described in Rothen et al. (2017). For a second batch of 15 isolates, paired-end libraries constructed by the Nextera XT DNA library prep kit (Illumina, San Diego, CA, United States) were sequenced on a MiSeq system (Illumina) using a 600-cycle MiSeq reagent kit v3 (Illumina). *De novo* assemblies were created using SeqMan NGen from the Lasergene genomics package version 12.1.0 (DNAStar, Madison, WI, United States) with standard settings. Comprehensive WGS information including accession numbers are provided in Supplementary Table S1.

### *In silico* Prediction of Capsular Serotype and Multi-Locus Sequence Typing

*In silico* capsular typing was performed as described by Sheppard et al. (2016). MLST was carried out using a custom R script, accessing the query references of the seven housekeeping genes from the PubMLST database^1^. Each defined CC consisted of a founder ST and its single-locus variants (SLV). Remaining ST that were double-locus variants (DLV) of founder ST were assigned to the corresponding CC and four ST (ST22, ST26, ST130, and ST616) that could not be attributed to a CC were defined as stand-alone ST.

### *In silico* Typing of GBS Surface Proteins

tBLASTn analyses were carried out for an *in silico* variant typing of five major GBS surface proteins (Supplementary Table S3). For variant typing of the laminin-binding protein (Lmb) and the surface immunogenic protein (Sip), one query sequence was used for BLAST and the identified protein variants assigned an allele number in decreasing order of frequency. Variant-specific protein sequences published by Creti et al. (2004) were used as query files for the alpha-like protein (Alp) gene family. For the surface protein gbs2018 (BibA), variant-specific query sequences described by Springman et al. (2009) were used. Distribution of pilus islands (PIs) types among the WGS was determined using representative sequences of the three described variants PI-1, PI-2a, and PI-2b (Martins et al., 2013).

### Core-Genome Phylogenetic Analysis

Automatic genome annotation of the WGS was performed with the Prokka software tool version 1.12 (Seemann, 2014), using a *Streptococcus* genus database. The core-genome phylogenetic relationships of the WGS were obtained using EDGAR version 2.2 (Blom et al., 2016). Briefly, the core-genome was defined by iterative pairwise comparison of the gene content of each of the selected genomes using the bidirectional best hits (BBH) with score ratio values as orthology criterion (Blom et al., 2016). For all calculations protein BLAST (BLASTp) was used with BLOSUM62 as similarity matrix (Altschul et al., 1990; Henikoff and Henikoff, 1992). Multiple alignments of each of the 867 orthologous gene set of the core genome were calculated using the MUSCLE software (Edgar, 2004), which equaled 690,132 genes in total. The resulting alignments were concatenated to one huge alignment (Talavera and Castresana, 2007), which consisted of 212,086,240 amino acid residues, 266,440 per genome. This alignment was used to construct a FastTree phylogeny (Price et al., 2009). Phylogenetic trees were visualized and edited using the interactive tree of life (iTOL) website (Letunic and Bork, 2016).

### *In silico* Molecular Weight Prediction of Ribosomal Subunit Proteins

The theoretical monoisotopic molecular weights of rsp were predicted using an in-house Python bioinformatics pipeline. Post-translational modifications, specifically N-terminal methionine loss and methylation, were taken into account. While a total of 17 rsp (S8-S10, S12, S13, S15, S16, S18, S19, S21, L6, L17, L21, L22, L30, L32, and L35) were found to display N-terminal methionine loss, no methylation was observed for any of the rsp. tBLASTn analyses were carried out for an *in silico* typing of the rsp in 796 GBS WGS. Based on the predicted 28 rsp masses in our collection, we assessed the variability of each mass (mass alleles) and defined unique combinations of mass alleles (rsp-profiles) across the WGS, taking into account the MALDI-TOF MS detection threshold of 400 ppm.

### GBS Isolates Used for MALDI-TOF MS Analyses

The 248 GBS isolates used in this study were obtained from four different sources: (i) 156 human isolates belonging to a set of *S. agalactiae* strains described by Huber et al. (2011). These both inpatient and outpatient samples were obtained and cultivated at the Aga Khan University Hospital in Nairobi, Kenya between January 2007 and June 2010; (ii) Seventy-nine samples from the International Livestock Research Institute (ILRI) isolated from camels in Kenya and Somalia (Fischer et al., 2013); (iii) Six GBS samples from cattle, isolated during 2009 in Switzerland by Prof. J. Frey from the University of Bern (unpublished); (iv) Seven human GBS reference strains were provided by Dr. H. Tettelin from the University of Maryland (Tettelin et al., 2005). More comprehensive information of all analyzed GBS isolates is provided in Supplementary Table S4.

### Bacteria Cultivation and Sample Preparation

GBS isolates were stored at -80°C prior to cultivation. After thawing the isolates on ice, bacterial material was plated on Columbia Sheep Blood Agar. The plates were then stored at 37°C in the incubator for overnight cultivation. Single colonies were transferred to a new agar plate using the four-quadrant streak method. After repeated overnight cultivation at 37°C*, S. agalactiae* colonies were harvested for sample preparation. The bacteria material was washed repeatedly in TMA buffer (10 mM Tris–HCl (pH 7.8), 30 mM NH_4_Cl, 10 mM MgCl_2_, and 6 mM 2-mercaptoethanol). In a next step, the bacterial cells were disrupted using a FastPrep FP120 bead beater in order to lay open intracellular proteins. To that end, the washed cells were transferred together with 0.1 mm glass beads to a micro tube. The mixture was agitated for multiple short time intervals (20s) at maximum speed, interrupted by cooling intervals (1 min) on ice. In a last step, protein fragments smaller than 3,000 Dalton (Da) were removed by filtering of the bacterial extract with Amicon^TM^ Ultra centrifugal devices. Lastly, the concentrated sample was mixed with the tenfold volume of ddH_2_O and 1 μl of the dilution was applied in quadruplicates on a MALDI-TOF steel target plate. The spotted samples were left to air dry at room temperature and consequently overlaid with a matrix consisting of a saturated solution of 10 mg sinapinic acid in 60% acetonitrile, 40% ddH_2_O and 1% TFA.

### MALDI-TOF MS Analyses

#### Instrument Setup

The MS measurements were carried out using a MALDI-TOF Mass Spectrometer Axima Confidence machine (Shimadzu-Biotech, Kyoto, Japan) with detection in the linear positive mode, allowing the interrogation of high molecular weight samples. The acceleration voltage was set by default to 20 kV with an extraction delay time of 200 ns and a laser frequency of 50 Hz. The analysis was carried out in the mass range between 4,000 and 25,000 Da. To ensure an even measurement covering the entire area of the sample spot, a netlike pattern of 100 equally distributed locations was defined. At each of these profiles ten consecutive laser shots were applied, adding up to 1,000 laser shots per sample spot. The ion gate was set at 3,950 Da and the pulsed extraction optimized at 20,000 Da. Each target plate was externally calibrated using the reference spectra of in-house cultured *Escherichia coli* strain DH5α.

#### Mass Spectra Processing and Internal Calibration

The individual mass fingerprints were averaged and the spectra further processed with the Launchpad 2.8 software (Shimadzu-Biotech, Kyoto, Japan). The advanced scenario setting was chosen for peak processing, with a defined peak width of 80 chans, smoothing filter width of 50 chans and baseline filter width of 500 chans. An adaptive voltage threshold, which roughly followed the signal noise level, was defined and the threshold offset and threshold response set to 0.008 and 1.000, respectively. Internal calibration with 800 ppm was carried out with MALDIquant (Gibb and Strimmer, 2012), using 10 rsp masses (3 mass alleles of L6, 2 mass alleles of L36 and S12, 1 mass allele of L14, L29, and S15) that altogether display mass values distributed over a wide mass range (4,425–19,293 Da). An ASCII file containing the recalibrated protein mass values and corresponding intensities was automatically generated for every mass spectrum.

#### Classification of Mass Spectra

The mass spectra were classified using a custom Python script (Supplementary Data Sheet S2). Briefly, all mass spectrum peaks were queried against the *in silico* predicted mass alleles of 28 rsp and the thereby generated sequence of mass alleles matched against the reference library containing the 62 defined rsp-profiles. A mass list was assigned an rsp-profile identification (ID) if (i) there was one single top matching reference and (ii) if at least 24 rsp masses could be detected. An isolate was assigned a final rsp-profile ID if (i) at least two of the four technical replicate mass lists were assigned the same rsp-profile and (ii) if there was no contradicting match with a different rsp-profile in the other technical replicate mass lists. If a specific rsp was missing in all mass lists considered for the final ID of an isolate, a warning message was generated, indicating the possibility of a new rsp-profile not yet contained in the database.

#### Confirmation of Inter-Laboratory Reproducibility of Our Method

In order to confirm the inter-site transferability and reproducibility of our method, additional MALDI-TOF MS analyses were performed in an independent laboratory. Eight GBS isolates were cultivated and pre-processed following our established protocol. The MALDI-TOF measurements were carried out on a Microflex machine (Bruker Daltonics, Bremen, Germany), with the instrument parameter settings adjusted for the use of sinapinic acid. Spectra post-processing, internal calibration, rsp prediction and classification was carried out in an automated way using our custom R and python scripts as described above. WGS data of the eight GBS isolates were available and used *in silico* to confirm the molecular masses of the 28 rsp.

## Data Availability

Whole genome sequences generated for this study can be found in NCBI GenBank, PRJNA490650. The script and dependencies used for the classification of GBS mass lists, well as any future releases of the *in silico* workflow, are available on GitHub (https://github.com/JRothen). Detailed information regarding the scripts used for the *in silico* prediction of rsp molecular masses is available from the authors upon request.

## Author Contributions

CD, GV, MI, MT, and VP contributed to the design of the study. JR and DN performed the MALDI-TOF MS analyses. JP, FF, and JR conducted the *in silico* parts of the analyses. JP, JR, DN, and CL performed DNA extraction and whole-genome sequencing. JB carried out the core-genome phylogenetic analyses. JR, VP, and CD wrote the manuscript. All the authors read and approved of the final version of the manuscript.

## Conflict of Interest Statement

FF, GV, and VP are employed by Mabritec AG. The remaining authors declare that the research was conducted in the absence of any commercial or financial relationships that could be construed as a potential conflict of interest.

## Footnotes

1 https://pubmlst.org/sagalactiae/
