# Supplementary material for: Subspecies Typing of Streptococcus agalactiae Based on Ribosomal Subunit Protein Mass Variation by MALDI-TOF MS
Source: Front Microbiol. 2019 Mar 11;10:471. doi: 10.3389/fmicb.2019.00471 (PMC6421976; doi:10.3389/fmicb.2019.00471)
Supplement: Supplementary file 3 [file Data_Sheet_1.pdf]

Supplementary Information for

Subspecies typing of *Streptococcus agalactiae* based on ribosomal subunit protein mass variation by MALDI-TOF MS

Julian Rothen; JR*, Joël F. Pothier; JFP, Frédéric Foucault; FF, Jochen Blom; JB, Dulmini Nanayakkara; DN, Carmen Li; CL, Margaret Ip; MI, Marcel Tanner; MT, Guido Vogel; GV, Valentin Pflüger; VP*, Claudia A. Daubenberger; CAD

*Corresponding authors:

Julian Rothen
Email: julian.rothen@unibas.ch

Valentin Pflüger
valentin.pflueger@mabritec.com

This PDF file includes:

Supplementary Figures S1, S2 and S3
Captions for Supplementary Tables S1, S2, S3, S4 and Supplementary Data Sheet S1.

Other supplementary materials for this manuscript include the following:

Supplementary Tables S1, S2, S3, S4 (one file) and Supplementary Data Sheet S1.

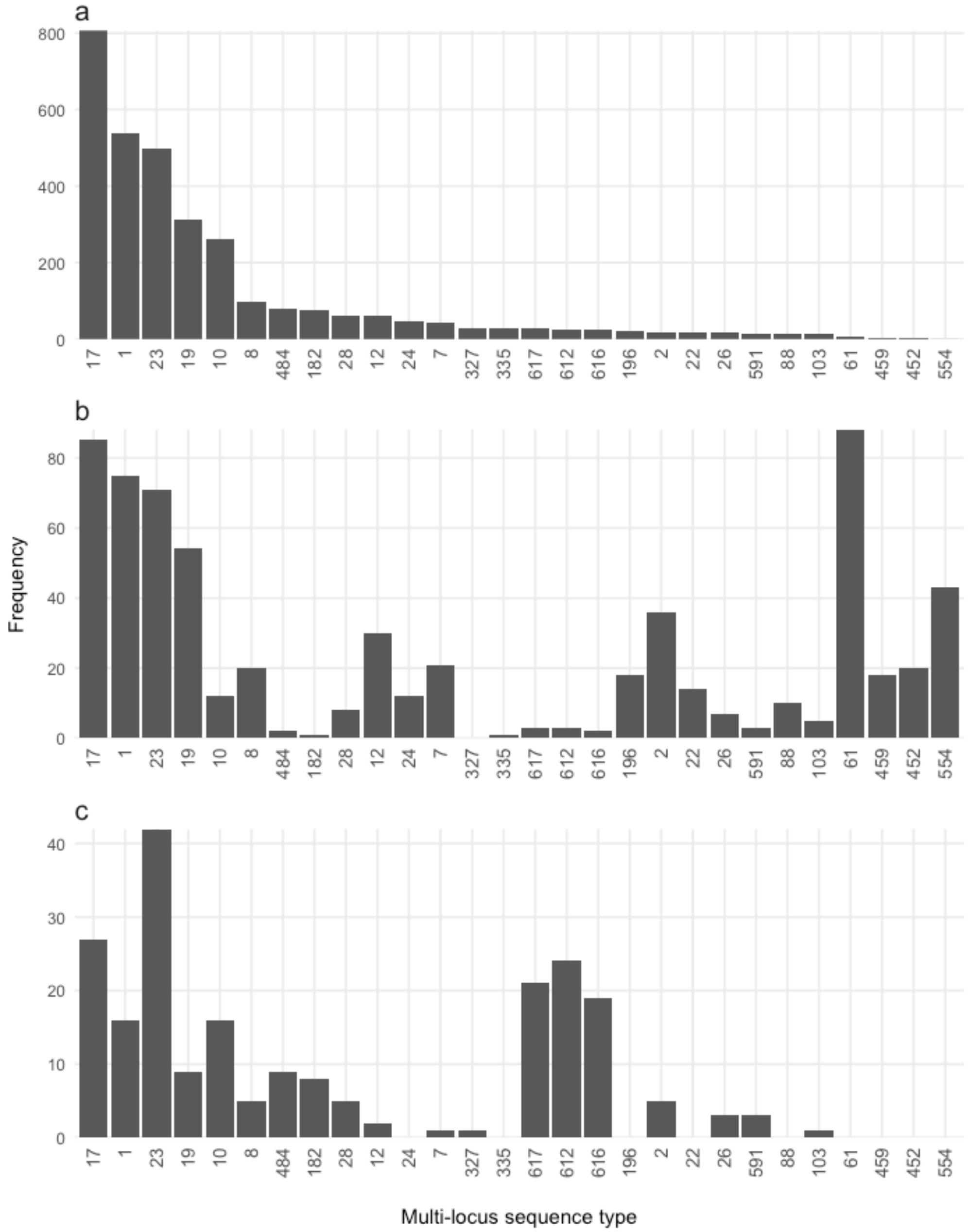

Supplementary Figure S1. Frequencies of 28 multi-locus sequence types in: (a) global Group B *Streptococcus* (GBS) population as assessed on 05-10-2017 on PubMLST (Jolley and Maiden, 2010); (b) 796 GBS whole genome sequences analyzed in this study; (c) 248 GBS isolates analyzed by MALDI-TOF MS in this study.

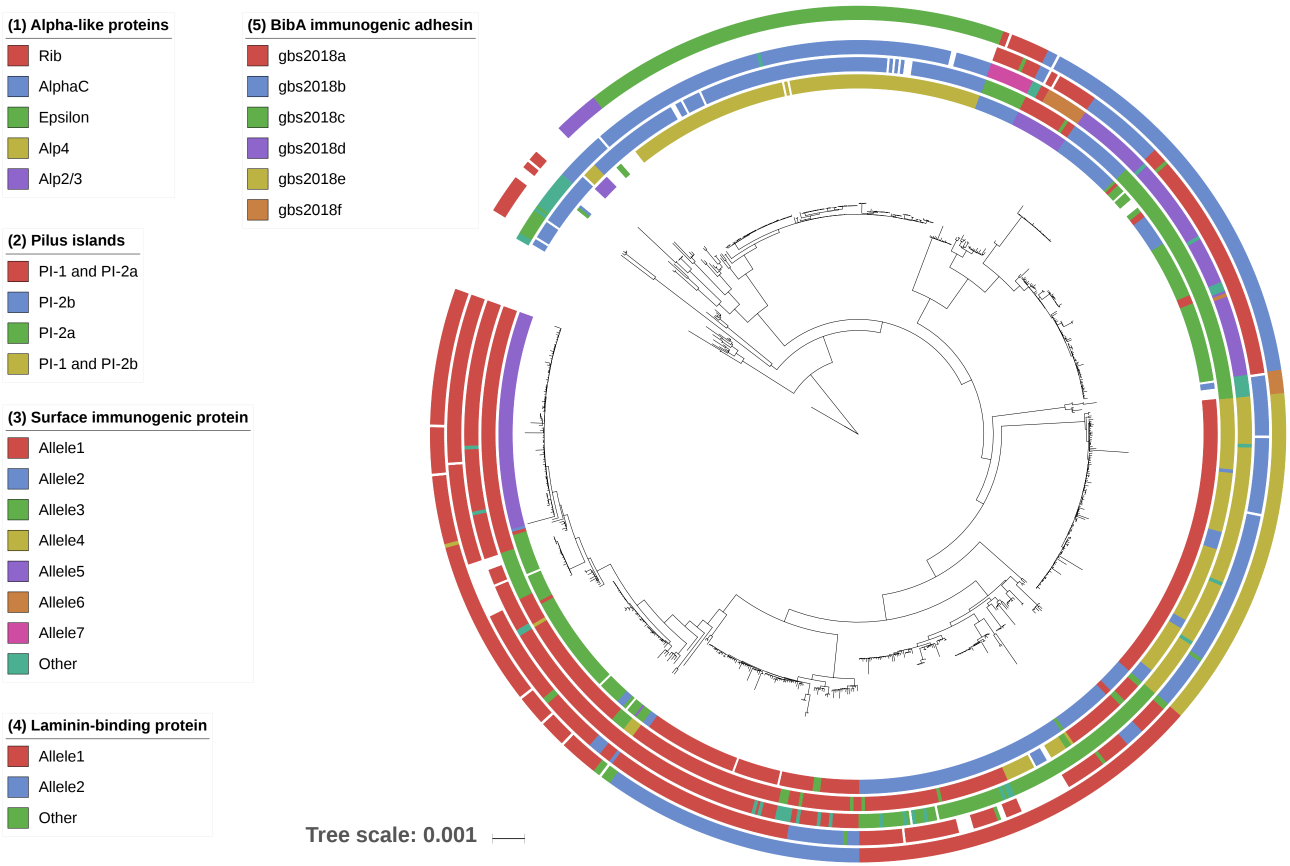

Supplementary Figure S2. Phyolgenetic tree **(**FastTree) based on core-genome analysis of 796 Group B *Streptococcus* (GBS) whole genome sequences. Each GBS strain is annotated with its *in silico* determined surface protein allelic variants. From inner circle (1) to outer circle (5): Alpha-like protein (Alp) family, pilus islands, surface immunogenic protein (Sip), laminin-binding protein (Lmb) and Group B *Streptococcus* immunogenic bacterial adhesin (BibA). (Scale bar: nucleotide substitutions per site.

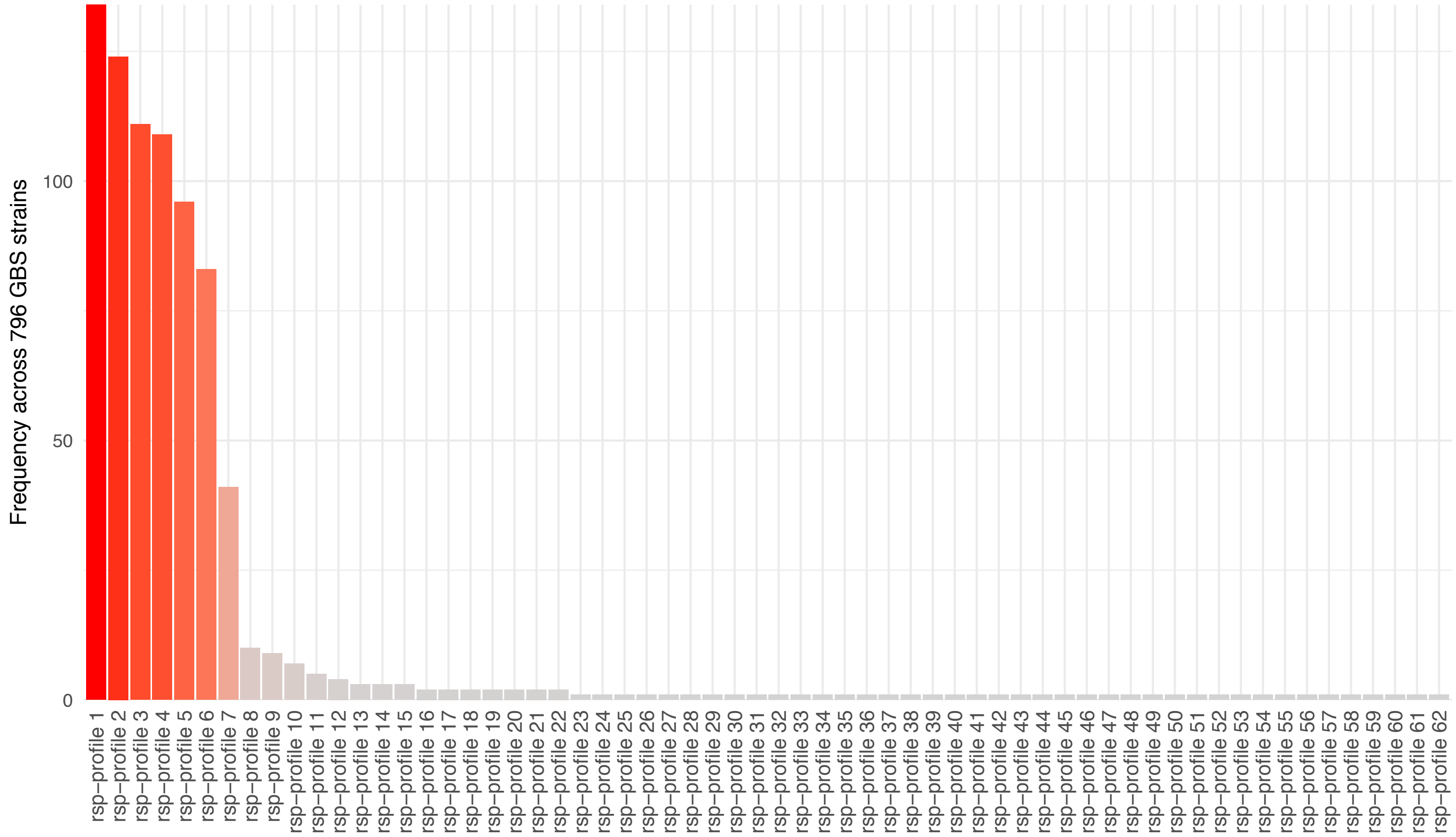

Supplementary Figure S3. Frequencies of ribosomal subunit protein (rsp) profiles across 796 Group B *Streptococcus* (GBS) strains. *In silico* prediction of molecular mass variability of 28 rsp revealed the occurrence of six dominat rsp-profiles (1-6) in the global GBS population.

Additional Supplementary Table S1 (separate file)

Metadata of 796 whole genome sequenced Group B *Streptococcus* (GBS) strains used in this study. ST: multi-locus sequence type; SLV: single-locus variant; CC: clonal complex; Alp: alpha-like protein; Sip: surface immunogenic protein; Lmb: laminin-binding protein; BibA: group B *Streptococcus* immunogenic bacterial adhesin protein.

Additional Supplementary Table S2 (separate file)

Predictive value of the six dominant, MALDI-TOF MS assigned ribosomal subunit protein (rsp)-profiles 1-6 with respect to Group B *Streptococcus* (GBS) capsular serotype, clonal complex (CC) and surface protein variants.

Additional Supplementary Table S3 (separate file)

Protein queries used for *in silico* identification of major Group B *Streptococcus* surface protein variants.

Additional Supplementary Table S4 (separate file)

Metadata of 248 Group B *Streptococcus* (GBS) in-house isolates and 8 GBS isolates from an external laboratory that were subtyped by MALDI-TOF MS in this study.

Additional Supplementary Data Sheet S1 (separate file)

Python code used for automated classification of Group B *Streptococcus* mass spectra files.

References

Jolley, K. A., and Maiden, M. C. (2010). BIGSdb: Scalable analysis of bacterial genome variation at the population level. *BMC Bioinformatics* 11, 595. doi:10.1186/1471-2105-11-595.
