# Supplementary material for: Subspecies Typing of Streptococcus agalactiae Based on Ribosomal Subunit Protein Mass Variation by MALDI-TOF MS
Source: Front Microbiol. 2019 Mar 11;10:471. doi: 10.3389/fmicb.2019.00471 (PMC6421976; doi:10.3389/fmicb.2019.00471)
Supplement: Supplementary file 4 [file Data_Sheet_2.pdf]

```
# -*- coding: utf-8 -*-
"""
GBS ribo-MALDI Classification / Julian Rothen, February 2019
"""

## Define paths BEFORE running code ##

folder_path = ''
# path to directory containing files 'MolWeight_ribo_ascii' and
'62_rsp_binary'
folder_path2 = ''
# path to directory containing mass spectra ascii text files to be
analyzed

################################################################

import pandas as pd
import numpy as np
import itertools
import time
from collections import defaultdict
import glob

###############################################################


# master ascii contains all in silico predicted ribo masses

master_ascii = open(folder_path + 'MolWeight_ribo_ascii','r')

my_list = []

for line in master_ascii:
    my_list.append(line)

for entry in range(0,len(my_list)):
    my_list[entry] = my_list[entry].replace('\n','')

master_ascii.close()

df_master = pd.DataFrame()
df_master['ribo'] = np.array(my_list[7:86]) # ribosomal protein
names
df_master['mass'] = np.array(my_list[86:len(my_list)]) # predicted
mass

df_master['ribo'] = df_master['ribo'].str.replace('#SUB=','')
df_master['mass'] = df_master['mass'].str.replace(' 10','')
df_master['mass'] = pd.to_numeric(df_master['mass'],
errors='coerce')

# account for 400ppm error range

df_master['lower'] = df_master['mass'] - (df_master['mass']*0.0004)
```

```
df_master['upper'] = df_master['mass'] + (df_master['mass']*0.0004)

# For L36_2 and L36_1, increase error range


df_master.iloc[0,2] = 4420.0
df_master.iloc[0,3] = 4430.0
df_master.iloc[1,2] = 4445.0
df_master.iloc[1,3] = 4458.0

# binary contains 62 unique ribo mass combinations

binary = pd.read_csv(folder_path + '62rsp_binary.csv', sep = ';',
index_col= 'ID')


folder_path2 = '/Users/joules/Desktop/GBSProject/Validation/
Validation_FINAL_July2018/asciis_test/'
path_all = glob.glob(folder_path2 + '*.txt')

## define df to store results

df_matched_records = pd.DataFrame(columns=('Run','Sample','Ribos'))

## analyze ascii files one by one

for file in range(0,len(path_all)):

    master_ascii = open(path_all[file],'r', errors = 'ignore')

    ascii_masses = []
    sep= ' '

    for line in master_ascii:
        ascii_masses.append(line)

    master_ascii.close()

    start = ascii_masses[2].find('mabr_')
    end = ascii_masses[2].find('[c]', start)
    ascii_run = ascii_masses[2][start:end]

    start = ascii_masses[1].find('#Sample=')
    end = ascii_masses[1].find('#PSD=', start)
    ascii_sample = ascii_masses[1][start:end].replace('#Sample=','')

    ascii_masses = ascii_masses[7:len(ascii_masses)]

    ascii_intensities = list(ascii_masses)

    for entry in range(0,len(ascii_masses)):
        ascii_intensities[entry] =
ascii_intensities[entry].replace('\n','')
        ascii_intensities[entry] =
```

```
ascii_intensities[entry].split(sep, 1)[1]
        ascii_masses[entry] = ascii_masses[entry].replace('\n','')
        ascii_masses[entry] = ascii_masses[entry].split(sep, 1)[0]

    ascii_intensities = [float(i) for i in ascii_intensities]

    ######
    # ADD new Feature here: check for false positive hits and
exclude Variant(s) with lower intensity
    ######

    ascii_ribos = ['nomatch'] * len(ascii_masses)

    for mass in range(0,len(ascii_masses)):
        for entry in range(0,df_master.shape[0]):
            if float(ascii_masses[mass]) >=
np.asscalar(df_master.loc[entry,'lower']) and
float(ascii_masses[mass]) <=
np.asscalar(df_master.loc[entry,'upper']):
                ascii_ribos[mass] = df_master.loc[entry,'ribo']

    ascii_doubs = [elem[0:3] for elem in ascii_ribos]
    dupes = list(set([x for n, x in enumerate(ascii_doubs) if x in
ascii_doubs[:n]]))
    dupes.remove('nom')

    remove_ind = []

    for entry in dupes:

        # dupe-wise get list indices from ascii_ribos

        indices = [i for i, s in enumerate(ascii_ribos) if entry in
s]

        # identify indices with highest mass intensity

        high_int = []

high_int.append(ascii_intensities.index(max([ascii_intensities[i]
for i in indices])))

        # identify indices with low(er) mass intensity(ies)

        low_int = [x for x in indices if x not in high_int]

        remove_ind = remove_ind + low_int

        # use low_int indices to remove masses from ascii_masses

    for index in sorted(remove_ind, reverse=True):
        del ascii_masses[index]

    # match and extract ribos
```

```
    ascii_ribos = []

    for entry in range(0,df_master.shape[0]):

        for mass in range(0,len(ascii_masses)):

            if float(ascii_masses[mass]) >= 
np.asscalar(df_master.loc[entry,'lower']) and 
float(ascii_masses[mass]) <= 
np.asscalar(df_master.loc[entry,'upper']):
                ascii_ribos.append(df_master.loc[entry,'ribo'])


    # write to df with three columns (run, sample name and ribo 
list)

    df_matched_records.set_value(file, 'Run', ascii_run)
    df_matched_records.set_value(file, 'Sample', ascii_sample)
    df_matched_records.set_value(file, 'Ribos', ascii_ribos)

###############################################################
## define required functions

def combinations(iterable, r):
    pool = tuple(iterable)
    n = len(pool)
    if r > n:
        return
    indices = list(range(r))
    yield tuple(pool[i] for i in indices)
    while True:
        for i in reversed(range(r)):
            if indices[i] != i + n - r:
                break
        else:
            return
        indices[i] += 1
        for j in range(i+1, r):
            indices[j] = indices[j-1] + 1
        yield tuple(pool[i] for i in indices)


def list_duplicates(seq):
    tally = defaultdict(list)
    for i,item in enumerate(seq):
        tally[item].append(i)
    return ((key,locs) for key,locs in tally.items()
                            if len(locs)>1)


## define required objects

results_df = 
```

```
pd.DataFrame(columns=['Run','Sample','n_Ribo','list_Ribo','ID'])

full_length_profiles = pd.DataFrame(columns=list(range(0,28)))
full_length_profiles.insert(0,'ID',0)

for i in range(0, binary.shape[0]):
    full_length_profiles.loc[i,'ID'] = binary.index[i]
    full_length_profiles.iloc[i, 1:] =
binary.columns[binary.iloc[(i)]>0]


lower_t = 24

## code for matching

for a in range(0,df_matched_records.shape[0]):
    y = df_matched_records.loc[a,'Ribos']
    y2 = [elem[0:3] for elem in y]
    record_name = df_matched_records.loc[a,]

    # if n unique ribosomal subunits is below lower limit, no ID is
assigned:

    if len(set(y2)) < lower_t:
        pop_row = (results_df.shape[0]+1)
        results_df.loc[pop_row,'Run'] = record_name[0]
        results_df.loc[pop_row,'Sample'] = record_name[1]
        results_df.loc[pop_row,'n_Ribo'] = len(set(y2))
        results_df.loc[pop_row,'list_Ribo'] = y
        results_df.loc[pop_row,'ID'] = 'low mass count'

    # if n unique ribosomal subunits is above lower limit, proceed:

    else:
        doub_list = list()
        for b in range(0,len(list(list_duplicates(y2)))):
            for elem in list(list_duplicates(y2))[b][1]:
                doub_list.extend([elem])

        # if there are no ribosomal proteins with multiple hits
(stored in doub_list):

        if len(list(np.array(y)[doub_list])) == 0:

            # match the record against all possible same-length
combinations
            int_list = []
            for lineage in range(0,full_length_profiles.shape[0]):
                b2 =
pd.DataFrame(full_length_profiles.iloc[lineage,:]).transpose().value
s.tolist()[0][1:]
                int_list.append(len(set(y).intersection(b2)))

            max_hits = [i for i in range(len(int_list)) if
```

```
int_list[i] == max(int_list)]

            for hit in range(0, len(max_hits)):

                pop_row = (results_df.shape[0]+1) # choose row in
results df which is to be populated

                # populate results df

                results_df.loc[pop_row,'Run'] = record_name[0]
                results_df.loc[pop_row,'Sample'] = record_name[1]
                results_df.loc[pop_row,'n_Ribo'] = max(int_list)
                results_df.loc[pop_row,'list_Ribo'] = y
                results_df.loc[pop_row,'ID'] =
full_length_profiles.iloc[max_hits[hit],0]


        # if there are ribosomal proteins with multiple hits:
        # identify ribosomes with multiple hits and determine
possible alternative combinations

        else:
            comb1 = list(np.array(y)[doub_list])
            dupes = list(set([x for n, x in enumerate(y2) if x in
y2[:n]]))
            comb2 = list(combinations(comb1,len(dupes)))
            comb3 = [s for s in comb2 if len(set([elem[:3] for elem
in s])) == len(dupes)]
            comb4 = [s for s in y if s not in comb1]

            # build empty df to store all alternative combinations

            df_names2 = []
            for d in range(1,len(comb3)+1):
                df_names2.append('record_' + str(d))
                dic2 = {name: pd.DataFrame() for name in df_names2}

            # loop through all combinations and store combinations

            for e in range(0,len(comb3)):
                comb5 = [s for s in y if s in comb4 or s in
comb3[e]]
                dic2[df_names2[e]] = pd.DataFrame(comb5).transpose()

                # one by one, match combinations against full length
unique profiles

            for comb in range(0, len(df_names2)):

                b1 = dic2[df_names2[comb]].values.tolist()[0]
                int_list = []

                for lineage in
range(0,full_length_profiles.shape[0]):
```

```
                    b2 =
pd.DataFrame(full_length_profiles.iloc[lineage,:]).transpose().value
s.tolist()[0][1:]
                    int_list.append(len(set(b1).intersection(b2)))


                # get index of Lineages with highest intersection
                max_hits = [i for i in range(len(int_list)) if
int_list[i] == max(int_list)]

                for hit in range(0, len(max_hits)):

                    pop_row = (results_df.shape[0]+1) # choose row
in results df which is to be populated

                    # populate results df

                    results_df.loc[pop_row,'Run'] = record_name[0]
                    results_df.loc[pop_row,'Sample'] =
record_name[1]
                    results_df.loc[pop_row,'n_Ribo'] = max(int_list)
                    results_df.loc[pop_row,'list_Ribo'] = b1
                    results_df.loc[pop_row,'ID'] =
full_length_profiles.iloc[max_hits[hit],0]


## for multiple hits per Run in results_df, keep only highest
matches (n_Ribo)

idx = results_df.groupby(['Run'])['n_Ribo'].transform(max) ==
results_df['n_Ribo']
results_df = results_df[idx].reset_index()

##############################################################
results_df2 = results_df.copy()

results_df2['AssignedID'] = ''


def IDassign(x):
    if len(x.ID.dropna().unique()) == 1:
           return x.ID.dropna().unique()[0]

    if len(x.ID.dropna().unique()) > 1:
             return '_'.join(list(x.ID.dropna().unique()))

    if len(x.ID.dropna().unique()) == 0:
        return 'no ID'


run_ids = list(results_df2['Run'].unique())
final_ID =
pd.DataFrame(columns=('Run','Sample','n_Ribo','list_Ribo','ID','ribo
_missed'))
```

```
for run in range(0, len(run_ids)):
    final_ID.loc[run,'Run'] = run_ids[run]
    final_ID.loc[run,'Sample'] = results_df2.Sample[results_df2.Run
== run_ids[run]].unique()[0]
    final_ID.loc[run,'ID'] =
IDassign(results_df2.loc[results_df2.Run == run_ids[run],])
    final_ID.loc[run, 'n_Ribo'] = results_df2.loc[results_df2.Run ==
run_ids[run],].iloc[0,3]
    final_ID.loc[run, 'list_Ribo'] = results_df2.loc[results_df2.Run
== run_ids[run],].iloc[0,4] # not correct for multi-hits

    l1 = df_matched_records.loc[df_matched_records.Run ==
run_ids[run],'Ribos'].values[0]
    l2 = [elem[0:3] for elem in l1]
    l3 = [elem[0:3] for elem in df_master.ribo]

    final_ID.loc[run,'ribo_missed'] = list(set(l3) - set(l2))


final_ID.to_excel(folder_path + 'output.xlsx', encoding= 'utf-8')
```
